# Supporting people with visual impairment

**Published:** 2013

## Communication

Here are some techniques to keep in mind when meeting with a patient with visual impairment.

- Introduce yourself and say your name
- Use the name of the person so they know you are talking to them.
- Face the person and talk to them, not the person accompanying them.
- Be specific in giving directions: say ‘left’ or ‘right’ rather than pointing.
- Identify the room you are in if the person cannot see well enough to recognise their surroundings.
- Identify and name any other people in the room or involved in the consultation.
- Read out written information, including rights to treatment and associated risks.
- If the person moves to a new location, tell them who is in the room and offer to describe the environment.
- Do not leave the person alone in the centre of a room. Make sure they can touch a table, chair, or wall to maintain orientation to their surroundings.

## Children's education

There are different models for educating children with visual and other impairments, which include educating children in ‘special schools' (schools for children with specific impairments), and educating children in a mainstream school (known as ‘inclusive education'). The comments below apply equally well to any model, and eye care workers may wish to spend time talking to parents about them.

- All children have a right to education.
- Education will give a child more opportunities in the future.
- It is important that children with disabilities spend time with their peers to make friends, gain independence, and develop a sense of belonging in the community.
- Children with low vision will need regular and ongoing assessment to check their refraction and whether they are using the most appropriate low vision interventions. The interventions may need to change as the child grows older, and as his or her educational demands increase. For example, the font size in school books will get smaller as the child progresses through primary school.

*With thanks to Clare Gilbert, Co-director, International Centre for Eye Health, and the authors of Disability Inclusive Practices in Eye Health. CBM 2011*. www.cbm.org/disability-inclusive-eye-health

**FROM THE FIELD: Mobility impairments and access**

**Figure:**
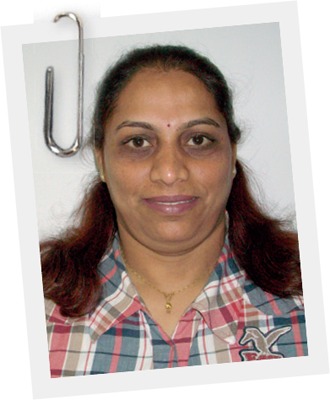
**Nagarathna**, CBM, Country Director: Sri Lanka, describes how she encouraged Joseph Eye Hospital in Sri Lanka to be more accessible. Nagarathna has a mobility impairment, and uses crutches to move around.

Nobody with a disability had ever come to Joseph Eye Hospital to work with them on increasing accessibility – it was all very new to them! I had to make several visits to the various hospital departments before they were able to understand the difficulties faced by people with mobility impairments.

Although the main entrance, wards, pharmacy, and optician's shop were accessible, the finance, administrative and management departments were not. But this is important: people with mobility impairments may want to discuss their fees, or need to talk about problems they are having.

When we started to talk about how the hospital could become more accessible without making big infrastructure changes, the idea no longer seemed so daunting and the hospital team were keen to make the necessary changes.

Some low-cost ways to improve access for people with impaired mobility include:

- Building a proper ramp with a railing – not too steep.
- Having at least one toilet that is accessible to someone in a wheelchair: a wide door, a western commode, a hand rail, and a low basin.
- Using non-slip flooring that is safe for people using crutches or calipers.
- Creating a section of the reception desk that is low enough so someone in a wheelchair can see the receptionist (and be seen by them).

Finally, I think it is important that eye clinics and non-governmental organisations employ people with disabilities in different capacities – this sets a good example and helps to ensure that the needs of disabled people will be met.

